# Bovine Coronavirus and the Associated Diseases

**DOI:** 10.3389/fvets.2021.643220

**Published:** 2021-03-31

**Authors:** Anastasia N. Vlasova, Linda J. Saif

**Affiliations:** Center for Food Animal Health Research, College of Food, Agricultural and Environmental Sciences, The Ohio State University, Wooster, OH, United States

**Keywords:** cattle, bovine coronavirus, respiratory, bovine respiratory disease complex, diarrhea, enteric, wild ruminants

## Abstract

Coronaviruses (CoVs) possess the largest and most complex RNA genome (up to 32 kb) that encodes for 16 non-structural proteins regulating RNA synthesis and modification. Coronaviruses are known to infect a wide range of mammalian and avian species causing remarkably diverse disease syndromes. Variable tissue tropism and the ability to easily cross interspecies barriers are the well-known characteristics of certain CoVs. The 21st century epidemics of severe acute respiratory CoV (SARS-CoV), Middle East respiratory CoV and the ongoing SARS-CoV-2 pandemic further highlight these characteristics and emphasize the relevance of CoVs to the global public health. Bovine CoVs (BCoVs) are betacoronaviruses associated with neonatal calf diarrhea, and with winter dysentery and shipping fever in older cattle. Of interest, no distinct genetic or antigenic markers have been identified in BCoVs associated with these distinct clinical syndromes. In contrast, like other CoVs, BCoVs exist as quasispecies. Besides cattle, BCoVs and bovine-like CoVs were identified in various domestic and wild ruminant species (water buffalo, sheep, goat, dromedary camel, llama, alpaca, deer, wild cattle, antelopes, giraffes, and wild goats), dogs and humans. Surprisingly, bovine-like CoVs also cannot be reliably distinguished from BCoVs using comparative genomics. Additionally, there are historical examples of zoonotic transmission of BCoVs. This article will discuss BCoV pathogenesis, epidemiology, interspecies transmission, immune responses, vaccines, and diagnostics.

## Introduction

Coronaviruses (CoVs) are enveloped viruses with the largest RNA genome (26.4–31.7 kb) that belong to the subfamily of *Coronavirinae* within the family *Coronaviridae*, order *Nidovirales* (1). Currently, CoVs are classified into four genera: *Alphacoronavirus, Betacoronavirus, Gammacoronavirus*, and *Deltacoronavirus*, with alpha- and betacoronaviruses represented by mammalian CoVs, while all avian CoVs are members of the other two genera (1).

Coronaviruses infect a wide diversity of mammalian and avian species causing respiratory, enteric, neurologic and hepatic disorders (1). The 21st century has already provided abundant and well-documented evidence of the ability of CoVs to quickly adapt to new hosts and ecological niches. Examples of expansion of host and geographical ranges include the severe acute respiratory coronavirus (SARS-CoV) pandemic, as well as the ongoing Middle East respiratory coronavirus (MERS-CoV) epidemic and the SARS-CoV-2 pandemic; emergence of porcine epidemic diarrhea virus in both Americas, and porcine deltacoronavirus emergence and spread in Asia followed by its spread to the US (2–6). This ability to quickly adapt to novel hosts and ecological niches are attributed to the high mutation rate (number of mutations/genome/round of replication) caused by relatively low fidelity of the viral RNA polymerase (7), the large genomes of CoVs (8), and the high frequency of homologous recombination events during RNA replication well-documented for several porcine, feline and canine coronaviruses (9–13). CoVs are the only known RNA viruses that evolved a mechanism for proofreading their genomes (via activities of non-structural proteins 10–14) allowing them to escape lethal error catastrophe events (14) and to generate and maintain highly diverse and viable quasispecies pools.

Several human CoVs (HCoVs) associated with common colds and acute gastroenteritis, HCoV-229E, HCoV-HKU1, HCoV-NL63, HCoV-OC43, and human enteric CoV 44 (HECoV-44) were recognized (15) even before modern tools for molecular characterization and diagnostics were available and the epidemic/pandemic nature of CoVs was widely appreciated. Some of them were of suspect zoonotic origin (15, 16) including spillover from cattle (HCoV-OC43 and HECoV-44) (15, 17, 18).

Bovine coronavirus (BCoV) is a pneumoenteric virus that belongs to the species *Betacoronavirus 1* (subgenus *Embecovirus*) of the *Betacoronavirus* genus along with wild ruminant CoVs, porcine hemagglutinating encephalomyelitis virus, equine coronavirus, HCoV-OC43, HECoV-44, and canine respiratory coronavirus (1). Due to their close antigenic and genetic relatedness, *Betacoronavirus 1* species members appear to be host-range variants originating from the same parental virus as a result of multiple genetic recombination and interspecies transmission events (17, 19–21).

Bovine CoV particles are enveloped and pleomorphic, 65–210 nm in diameter (22). Bovine CoV particles possess 5 major structural proteins: the nucleocapsid protein (N, 50 kDa), the integral membrane (M, 25 kDa), the small membrane/envelope protein (E, 8 kDa), the haemagglutinin-esterase (HE, 120–140 kDa) and the spike (S, 190 kDa) (22). The latter consists of an S1 subunit that contains the dominant neutralizing epitopes and an S2 subunit that mediates viral membrane fusion. The HE acts as a receptor-destroying enzyme (esterase) to reverse hemagglutination. The N protein lies internal to the virus envelope and is associated with the viral RNA, the M spans the viral envelope while the S and HE project from the envelope. Additionally, 16 non-structural proteins (nsp1-16) have been identified in betacoronaviruses (23, 24). Like other enveloped viruses, BCoVs are sensitive to detergents and lipid solvents (including ether, chloroform) and are easily inactivated by most conventional disinfectants, formalin, and heat.

**Figure 1:**
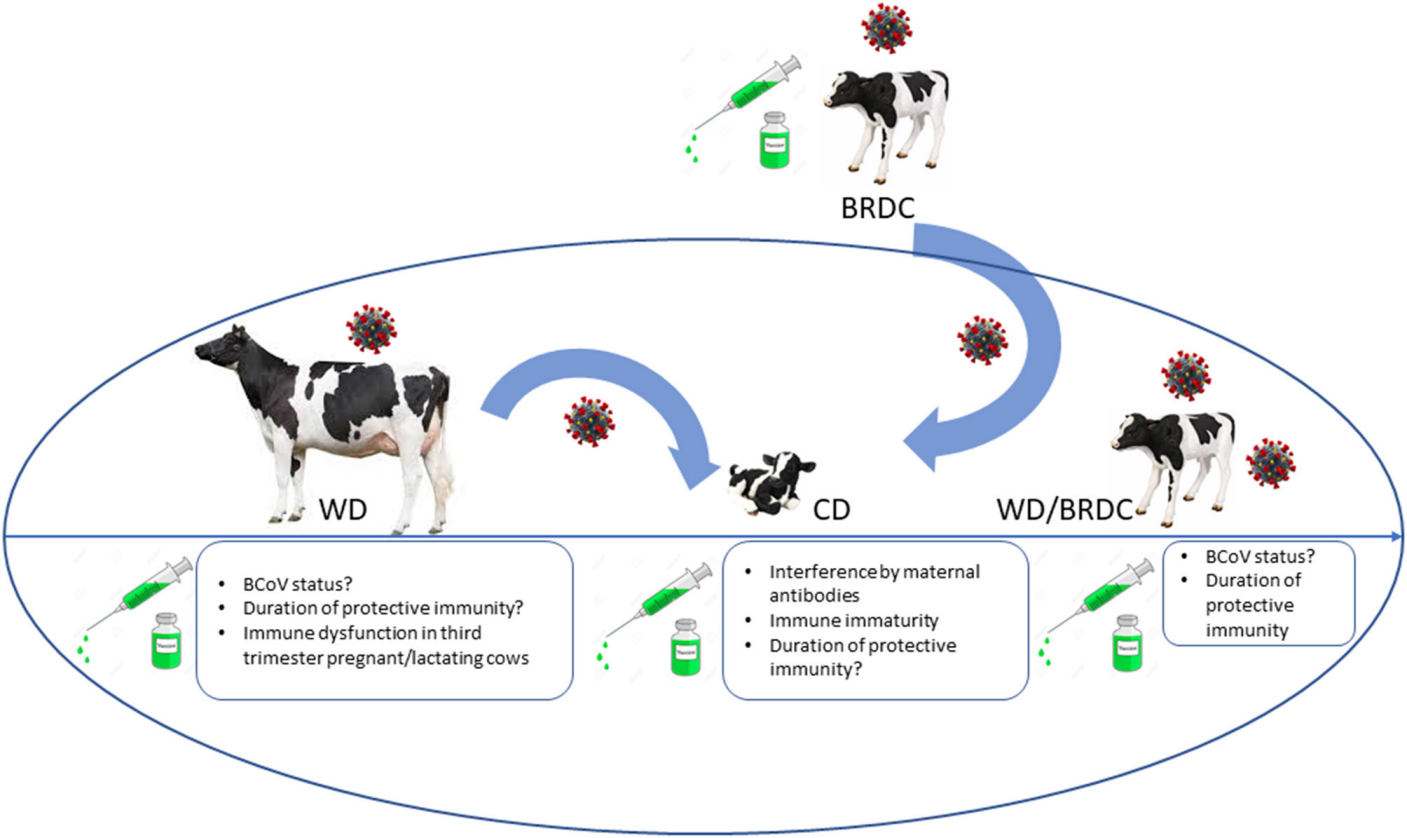
Summary diagram of different clinical syndromes associated with BCoV and challenges for successful vaccination associated with different ages/production status of cattle. CD, calf diarrhea; WD, winter dysentery; BRDC, bovine respiratory disease complex. Coronavirus image placed above animal represents potential carrier status. Rectangle boxes list unknown host and vaccine-associated factors that can result in suboptimal vaccine performance or lack of protection of cattle of different ages.

Bovine coronaviruses cause respiratory and enteric diseases in cattle and other ruminants but can be identified in the respiratory and intestinal tracts of healthy cattle (22, 25–27). BCoV is shed in feces and nasal secretions and is associated with 3 distinct clinical syndromes in cattle (27): (neonatal) calf diarrhea [(N)CD)] (22, 28), winter dysentery (WD) characterized by hemorrhagic diarrhea in adults (25, 28–30) and respiratory infections in cattle of different ages commonly as a part of the bovine respiratory disease complex (BRDC) or shipping fever of feedlot cattle (22, 26, 31, 32) (Figure 1). BRDC causes major economic losses to the beef and dairy cattle industries worldwide due to substantial morbidity and mortality. In North America, this complex represents the leading cause of morbidity and mortality in 6–10-month-old beef cattle after entry into feedlots (33).

**Figure 2:**
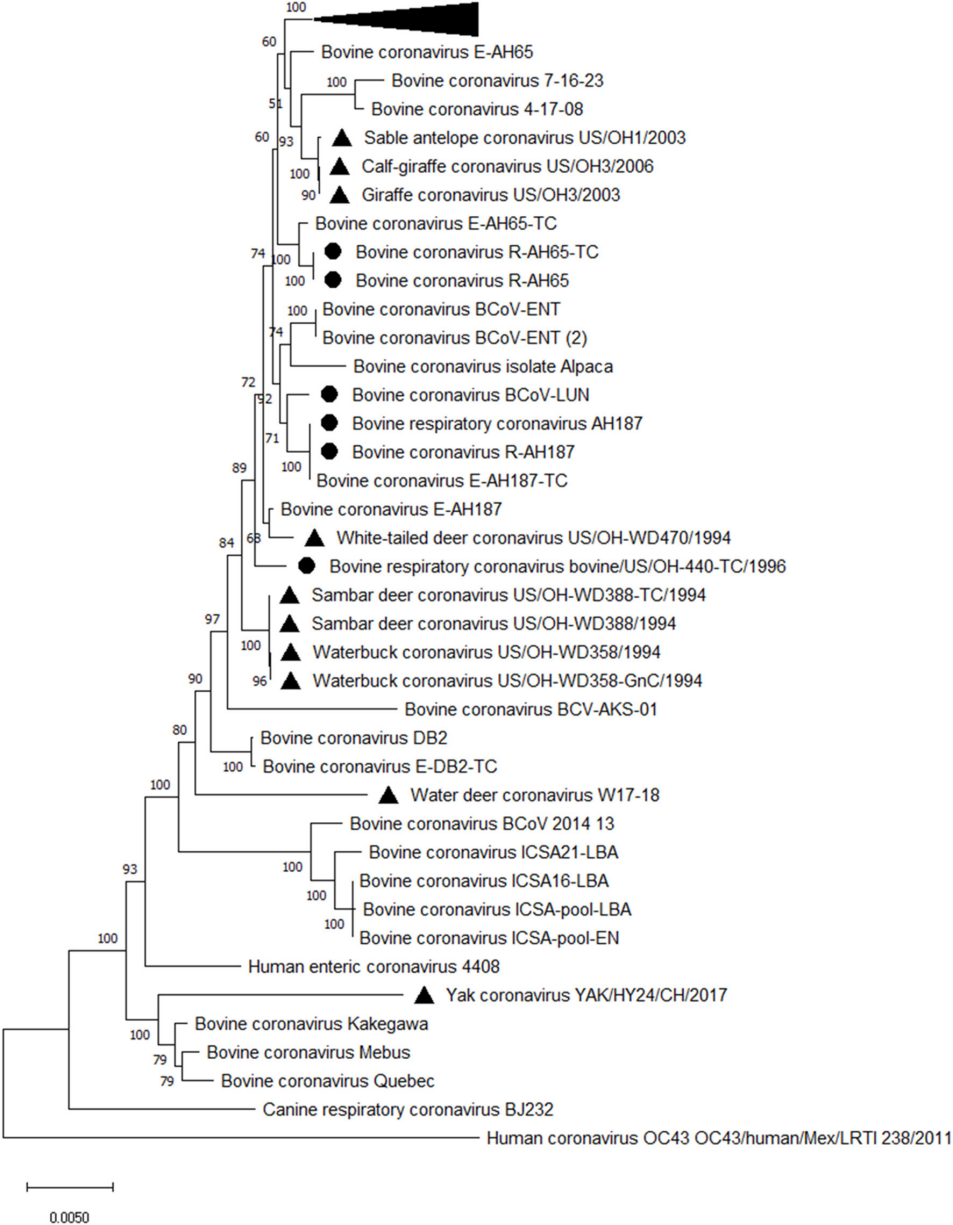
Phylogenetic analysis of complete genomes of enteric and respiratory BCoVs and bovine-like CoVs from wild ruminants. The evolutionary history was inferred by using the Maximum Likelihood method and General Time Reversible model. The tree with the highest log likelihood (−81,750.81) is shown. The percentage of trees in which the associated taxa clustered together is shown next to the branches. The tree is drawn to scale, with branch lengths measured in the number of substitutions per site. This analysis involved 53 nucleotide sequences. Evolutionary analyses were conducted in MEGA X (39). Black triangle markers are used for bovine-like CoVs isolated from wild ruminants, and black round markers are used to mark respiratory BCoVs. The collapsed branch includes a cluster of recent BCoV strains from Japan (35).

All BCoV isolates identified so far are shed in feces and nasal secretions and belong to a single serotype/genotype based on virus cross-neutralization and genotyping analyses regardless of clinical origin (27, 28, 34, 35). However, genotyping identified distinct sublineages and clusters based on the year and place of isolation but not on the disease type (19, 35–38) (Figure 2). Similarly, 2 to 3 subtypes of BCoV are recognized based on their biologic and antigenic characteristics identified in virus neutralization and ELISA [with monoclonal antibodies (MAbs)] tests without association with the different disease types (22, 26–28). While some studies identified mutations potentially associated with respiratory and enteric phenotypes (40–42); these findings have not been consistently confirmed by other groups in observational or experimental studies (36). Further, despite numerous genetic differences (point mutations and deletions) detected in the spike (S) gene between enteric and respiratory isolates or between BCoV and bovine-like CoVs from wild ruminants and humans, *in vivo* and *in vitro* experiments demonstrated high levels of cross-protection and cross-neutralization between such isolates (36, 43–48). Thus, no genetic or antigenic markers associated with the different disease manifestations have been identified, suggesting that the latter may result from the complex interplay between pathogens (CoV and other viruses or bacteria), host and environmental factors.

## Receptors and Attachment Factors

BCoV attaches to N-acetyl-9-O-acetylneuraminic acid (Neu5,9Ac_2_) through HE and S proteins to initiate infection (49–51). BCoV attachment is blocked by acetylesterase or neuraminidase treatment and can be restored by resialylation. It is further hypothesized that after the initial binding to sialic acid (SA)-containing receptors, the BCoV S protein may interact with a specific cellular receptor that leads to a conformational change and the viral-cell membrane fusion (52). A study demonstrated that similar to HCoV-OC43, BCoV preferentially used α-2,6-(SA) and not α-2,3-SA; however, this effect was less pronounced for BCoV compared with HCoV-OC43 (53). The same study has shown that BCoV (similar to canine respiratory CoV) employs human leukocyte antigen class I (HLA-I) as an entry receptor. HLA class I molecules that belong to the immunoglobulin superfamily consist of three α chains linked with a β2-microglobulin molecule that adopt a standard immunoglobulin-like fold (54). The receptor-binding (lectin) and receptor-destroying (esterase) domains of the HE protein also play important roles for viral entry (55); although, these interactions may be weaker than for the S protein because HE is a less efficient hemagglutinin. Additionally, heparan sulfate was identified as an alternative attachment factor, but this may be related to the cell culture adaptation, not affecting the *in vivo* infection and pathogenesis (53).

Histo-blood group antigens (HBGAs) are known to play a role in cell attachment and pathogenesis of other betacoronaviruses, including SARS-CoV and SARS-CoV-2 (56, 57). Specifically, studies indicate that blood group A individuals are at higher risk of infection suggesting that HBGAs may serve as additional cellular receptors. It is currently unknown if BCoV can utilize HBGAs as cell entry receptors, which necessitates further research in this direction to understand the mechanisms of emergence of BCoV-like CoVs (such as HCoV-OC43 and HECoV-4408) into human population.

Overall, the complex interactions between BCoVs, their cellular receptors and mucosal microbiota need to be comprehensively evaluated to improve our understanding of BCoV epidemiology, pathogenesis and interspecies transmission.

## Epidemiology and Pathogenesis of Respiratory and Enteric Bovine Coronaviruses

Bovine coronavirus is widespread in cattle of all ages, resulting in economic losses to the beef and dairy industry throughout the world. The virus presence has been confirmed on all continents, and seroprevalence studies demonstrate that over 90% of cattle have been exposed to BCoV during their lifetime. Moreover, BCoVs are commonly identified in the respiratory and intestinal tracts of healthy and diseased cattle (58). Recent evidence suggests that BCoV can persist in colostrum-deprived calves or colostrum-fed calves with repeated nasal shedding (59) and detectable BCoV antibodies for up to 3 years suggesting that active immune response does not always result in viral clearance (60).

### Enteric BCoV Infections

After its accidental discovery in 1972 by Mebus et al. at the University of Nebraska (61), the virus was quickly isolated and characterized (62), and soon after recognized as a common cause of calf diarrhea (CD) (63). The virus plays a major role in the development of CD during the first 3 weeks of life in both dairy and beef cattle herds (27, 64). The disease results from the virus infecting both the small and large intestines, destroying villi and leading to severe, sometimes bloody, diarrhea and high mortality (65–67). Virus replication initially occurs in different sections of the small intestine subsequently spreading throughout the large intestine and causing a malabsorptive diarrhea. BCoV-infected intestinal epithelial cells (IECs) die, slough off, and are replaced by immature IECs. Stunted and fused small intestinal villi as well as atrophied colonic ridges are observed during pathological examination (65, 66, 68). The compensatory crypt hyperplasia and increased fluid secretion further exacerbates diarrhea (69). The loss of the mature IECs capable of absorption and digestive enzyme secretion greatly diminishes the absorptive, metabolic and secretory capacity of the intestinal tract (22, 69).

Continual feeding often provides more nutrients than the damaged small intestinal epithelium can absorb (70). This in turn leads to undigested nutrient fermentation in the large intestine, increased fluid accumulation, bacterial overgrowth and overproduction of organic acids, aggravating diarrhea (69, 71). Altogether, it leads to dehydration, metabolic acidosis and electrolyte imbalance (due to sodium, chloride, potassium, and bicarbonate loss) (72). While these pathological changes and the ensuing disease are most severe in younger animals, BCoV infection also contributes to winter dysentery (WD) development in adult dairy cattle that causes a dramatic decrease in milk production and significant economic losses (25, 27, 73).

BCoV causes enteritis in both dairy and beef herds, with most cases occurring within the first 30 days of life (22, 61, 64, 74). BCoV incidence in naturally occurring outbreaks of diarrheal disease reportedly may vary from 15 to 70% (68, 75, 76). CD may occur as early as 24 h of age in colostrum-deprived calves and as late as 5 months of age (59, 68, 77). Soon after becoming infected, calves begin shedding high amounts of BCoV (≤10^9^ viral particles/ml of feces) which can continue for up to 2 weeks; while during the convalescent phase recovered calves generally shed lower amounts for several weeks (78). While BCoV is detected in the feces of both diarrheic and healthy calves, diarrheic calves tested positive more often (incidence: 8–69%) than healthy ones (incidence: 0–24%) (68, 69, 75). Low-level intermittent BCoV shedding can be observed in >70% of healthy cows despite the presence of serum and intestinal BCoV-specific antibodies (76, 79). Because BCoV is more stable at lower ambient temperature and reduced ultraviolet light levels, BCoV shedding rates increase by 50–60% during the winter months (22, 80) likely contributing to WD development in adult cattle (29). BCoV shedding also increased by 65% at parturition, and by 71% 2 weeks postpartum due to immunological and hormonal perturbations in cows (80). Thus, calves born to BCoV-positive cows have a significantly higher risk of developing diarrhea due to periparturient exposure to the contaminated perineum, teats, and the calving area (81).

Besides being an important enteric pathogen of cattle, BCoVs are capable of interspecies transmission and causing disease in adoptive/spillover hosts. For example, HECoV-4408 from a child with acute diarrhea is closely related to BCoV genetically and antigenically and is suggested to be a BCoV variant (17). It was confirmed that it can infect and cause diarrhea in gnotobiotic calves as well as induce complete cross-protective immunity against the virulent BCoV-DB2 enteric CD strain (47). Based on complete genome analysis, it is hypothesized that porcine hemagglutinating encephalomyelitis virus and HCoV-OC43 have evolved from ancestral BCoV strains at some point in the past (42). This analysis provides evidence that CoVs of bovine/ruminant origin can become endemic in adoptive/spillover hosts. Enteric BCoVs (bovine-like CoVs) can also infect dogs (experimentally) and various domestic and wild ruminant species (naturally and experimentally), causing clinical (82) or subclinical infections and seroconversion (83, 84). These data raise questions of whether dogs or wild ruminants could also be a reservoir for bovine-like CoVs transmissible to cattle, or conversely, if cattle can transmit CoVs to dogs, other ruminant species and humans. Overall, the existing evidence indicates that BCoVs are associated with serious and economically significant disease.

### Respiratory BCoV Infections

In 1982 Thomas et al. was first to identify BCoV as one of contributing agents in calf pneumonia (85). Subsequently, numerous investigators have confirmed that enteric and respiratory BCoVs are members of the same quasispecies (86), despite genotypic and antigenic differences between individual isolates (26). Since 1995, the role of respiratory BCoV in BRDC development and reduced growth performance in feedlot cattle has been increasingly recognized (26, 27, 31, 87–90). Currently, respiratory tract infections in growing and feedlot calves are frequently attributed to BCoV (26, 27, 91, 92) and can manifest as mild respiratory disease (coughing, rhinitis) or pneumonia in 2–6-month-old calves (26, 27). A study demonstrated high respiratory BCoV nasal and fecal shedding rates of 84 and 96%, respectively (90). In another study, testing cattle 3-day pre-arrival demonstrated that nasal shedding consistently preceded fecal shedding (91). It was also demonstrated that many animals (61–74%) shed respiratory BCoV prior to shipping to feedlots (93). Fifty-eight to ninety-five percent of feedlot cattle seroconverted to BCoV by 3 weeks after arrival (87, 90, 94, 95). The widespread distribution of BCoV could be explained by two main factors: (1) shedding of high titers of the virus from respiratory and intestinal tracts (59, 92), and (2) existence of asymptomatic (carrier) animals within most herds. These carrier animals shed the virus in nasal secretions and feces and serve as a source of infection to neonates and other susceptible animals on the farm (59, 96).

BCoV seroprevalence studies that surveyed 135 Norwegian dairy herds have demonstrated that calves in BCoV-seropositive herds had an increased risk of respiratory disease development compared with BCoV-seronegative herds (97). Dairy calves in Ohio shed respiratory BCoV fecally and nasally with some of them developing diarrhea and/or respiratory disease (rhinitis) (59, 98). In a large feedlot study (*n* = 1,074 cattle), it was demonstrated that feedlot calves shedding respiratory BCoV nasally were more likely to have respiratory disease (1.6 times) and pulmonary lesions at slaughter (2.2 times) than animals that did not shed respiratory BCoV (31). In two subsequent studies, calves shedding respiratory BCoV nasally were 2.7 and 1.5 times more likely to have respiratory disease than those that did not shed (90, 91). In another report, nasal BCoV shedding or an antibody titer <20 correlated with increased risk of treatment for BRDC, while intranasal vaccination with BCoV vaccine mitigated this risk (99). Further, in 3 of 4 outbreaks in Italy, the classic signs of BRDC (dyspnea, fever, respiratory distress) were evident in 2–3-month-old calves positive for BCoV and negative for other respiratory viral pathogens, while presence of common bacterial pathogens of cattle (*Staphylococcus* spp. and *Proteus mirabilis* or *Mannheimia haemolytica*) was only confirmed in 2 of 4 outbreaks (92).

Despite this abundant circumstantial evidence derived from epidemiological studies and routine diagnostic testing for respiratory pathogens, the role of BCoV in the bovine respiratory disease complex (BRDC) has been debated for several decades (32). This is because attempts to experimentally reproduce clinical respiratory disease are often unsuccessful (45, 100–102) (Table 1). Of interest one out of the 4 studies that failed to reproduce clinical disease (Table 1) reported somewhat extensive pathological changes in the respiratory tract of the colostrum-deprived calves inoculated with KWD3 strain (102) (Table 1).

**Table 1:** Experimental studies to reproduce respiratory disease following BCoV inoculation.

**Study settings**	**Animal age, days**	**Animal number**	**Inoculation route**	**BCoV antigen in respiratory/intestinal tract**	**Respiratory tract pathology, disease**	**Intestinal tract pathology, disease**	**References**
**Experimental studies - no clinical disease**							
Cross-protection study in gnotobiotic calves using intestinal and respiratory BCoV material	1–24	9	Oral	Yes (upper resp. tract)/yes (large intestine+feces)	N/A, no clinical signs	N/A, diarrhea	(100)
	2–151	4	Intranasal+intratracheal	Yes (upper resp. tract)/yes (large intestine+feces)	N/A, no clinical signs	N/A, diarrhea	
Experimental inoculation of gnotobiotic and colostrum-deprived calves with a fecal BCoV isolate	3–50	11	Oral, intranasal, oronasal	Yes (upper resp. tract)/yes (feces)	N/A, no clinical signs	N/A, diarrhea	(101)
	25–63	7		Yes (upper resp. tract)/yes (feces)	N/A, no clinical signs	N/A, diarrhea	
Cross-protection study of BRCV, CD and WD isolates in colostrum-deprived and gnotobiotic calves	1–10	6	Oronasal	Yes (upper resp. tract)/yes (feces)	N/A, no clinical signs	N/A, diarrhea	(45)
	5–27	2	Oronasal	Yes (upper resp. tract)/yes (feces)	N/A, no clinical signs	N/A, diarrhea	
Experimental inoculation of colostrum-deprived calves with a WD strain	2–4	8	Oronasal	Yes (upper+lower resp. tract)/yes (small and large intestine)	Epithelial damage in nasal turbinates, trachea and lungs, and interstitial pneumonia, no clinical signs	Villous atrophy and crypt depth decrease in small and large intestine, respectively, diarrhea	(102)
**Experimental studies - clinical disease**							
Experimental inoculation of colostrum-deprived and colostrum-fed calves with a tracheal-organ culture supernatant containing BCoV	<7	7	Intranasal+transtracheal	Yes (upper+lower resp. tract)/yes (small and large intestine)	A few scattered areas of atelectasis, mild respiratory disease, cough and nasal discharge	N/A, diarrhea	(103)
Experimental inoculation of colostrum-deprived calves with attenuated Mebus and virulent Minnesota strains of BCoV	5	5	Oral	Yes (lungs)/yes (Crypts/peyers patches/feces)	N/A, Pneumonia, resp. distress	N/A, diarrhea	(78, 104)

In two studies, investigators were able to reproduce some features of respiratory disease of variable severity (Table 1) (78, 103, 104). In the first one, 7 colostrum-deprived or colostrum-fed calves received tracheal-organ culture supernatant containing BCoV (from a field case) and developed cough, nasal discharge, and diarrhea with a few scattered areas of atelectasis observed in lungs of 3 calves at post-mortem (Table 1). In another study, 3 colostrum-deprived calves were inoculated with a field virulent pneumoenteric BCoV (Minnesota); all of them developed diarrhea, 2 had pneumonia/respiratory distress, and 1 died (Table 1).

Thus, the BCoV-induced respiratory clinical disease and the associated pathological changes remain less defined than those for enteric BCoV (Tables 1, 2) (26, 27). The consensus opinion is that the respiratory disease and pathology may vary with BCoV strain, animal age, co-infections with other pathogens (from BRDC complex), weather and additional environmental stress factors (Table 2) (26, 27). The list of other viral and bacterial pathogens identified in association with BRDC is rather extensive and includes: parainfluenza virus, type 3, bovine respiratory syncytial virus, adenovirus, enterovirus, reovirus, influenza D virus, bovine viral diarrhea virus, bovine herpesvirus 1 and 4, *Mannheimia haemolytica, Pasteurella multocida, Histophilus somni, Mycoplasma bovis*, and *Trueperella pyogenes* (105). Multiagent experiments evaluated interactions between the respiratory pathogens and stress and emphasized their synergistic effects in the BRDC, but no such experiments have been reported for BCoV (32, 105, 106). A recent study has concurrently identified BCoV, *H. somni, M. haemolytica*, and *P. multocida* (but not other common BRDC pathogens) during a respiratory disease outbreak in pre-weaned beef calves emphasizing that these pathogens were likely the major contributors to the disease development (107). The multifactorial nature of BRDC makes treating it extremely challenging. For example, antibiotic treatment of animals with BRDC may lead to massive release of bacterial lipopolysaccharides (LPS) inducing proinflammatory cytokine response and further enhancing lung damage (27, 108) while treatment with corticosteroids can reduce the numbers of CD4 and CD8 T cells and certain cytokine levels compromising protective immunity and exacerbating disease (109, 110). Thus, current evidence suggests that BCoV alone may not be routinely associated with substantial infection/pathology of the lower respiratory tract, but likely is significant as a respiratory pathogen in the case of mixed infections and plays a major role in inciting the disease. However, experimental data on infection of SPF BCoV seronegative cattle with BCoV respiratory isolates is needed to better confirm the role of BCoV in respiratory disease.

**Table 2:** Respiratory BCoV pathogenesis in cattle of different ages.

	**Age group**		
	**Young calves**	**Adult cattle with WD**	**Feedlot calves with BRDC/shipping fever**
Respiratory disease	Coughing, fever, rhinitis, and inappetence, often with concurrent diarrhea	Seronegative cows: transient fever, mild cough, and serous mucopurulent discharge	Fever, dyspnea, inflammatory and necrotizing lung lesions leading to bronchopneumonia, weight loss, and death
		Seropositive cows: no disease	
Virus shedding	From both respiratory and intestinal tracts	Seronegative cows: from both respiratory and intestinal tracts	From both respiratory and intestinal tracts
		Seropositive cows: limited nasal shedding	
Pathological findings in respiratory tract	Sometimes: epithelial damage in nasal turbinates, trachea and lungs, interstitial pneumonia, atelectasis	N/A	Subacute exudative and necrotizing lobar pneumonia involving 50–80% of the lung volume. Histologic lung lesions were characterized as fibrinous, necrotizing lobar pneumonia, but with moderate to severe bronchitis and bronchiolitis
Comment	Experimental infections (if reproduce) result in milder disease	Disease is generally milder than in younger calves	Multifactorial disease: detection of *Mannheimia hemolytica* and *Pasteurella multocida* in addition to BCoV is highly common

## BCOV Interspecies Transmission

### BCoV-Like Infections of Wild and Domestic Ruminants

In the last several decades, numerous CoV strains sharing extensive biologic, antigenic and genetic similarities with BCoV (named therefore bovine-like CoVs) have been identified in the feces, intestinal contents or respiratory secretions of a diverse group of domestic and wild (captive or free-range) ruminant species (Table 3). Experimental inoculation of gnotobiotic (Gn) or colostrum-deprived calves demonstrated that many of the bovine-like CoVs were capable of efficient replication in these calves, produced enteric disease and generated cross-protective immune responses (48, 82). Additionally, 6.6, 8.7, and 13.3% of sera from white-tailed deer in Ohio, mule deer in Wyoming and caribous in Quebec, respectively, were seropositive for BCoV antibodies (82, 114) suggesting that natural infections are common.

**Table 3:** Bovine-like CoVs and antibodies (serum) identified in domestic and wild ruminants.

**Animal species**	**Location**	**Sample type**	**References**
Alpaca	USA (Oklahoma)	Feces	(111)
Alpaca	Peru	Feces	(112)
Alpaca	Peru	Intestinal lavage	(113)
Caribou/reindeer	Canada	Serum	(114)
Dromedary camel	USA (Wisconsin)	Feces	(115)
Dromedary camel	UAE (Dubai)	Feces	(116, 117)
Dromedary camel	Saudi Arabia	Nasal/rectal swab	(118)
Elk/Wapiti	Canada	Feces	(119)
Elk/Wapiti	USA (Kansas)	Feces	(120)
Goat	South Korea	Serum	(121)
Giraffe	USA (Ohio)	Feces	(48)
Himalayan tahr	South Korea	Feces	(122)
Llama, alpaca	USA (Oregon)	Feces	(123, 124)
Musk oxen	UK	Feces	(125)
Nyala	South Korea	Feces	(122)
Sable antelope	USA (Ohio)	Feces	(19)
Sambar deer	USA (Ohio)	Feces	(19, 28)
Sheep	USA (Idaho, Montana)	Feces	(126)
Sheep	Chile	Intestinal contents	(127)
Sheep, goat	Spain	Feces	(128)
Sheep, goat	Turkey	Intestinal contents	(129)
Sheep	Sweden	Serum	(130)
Sika deer	Japan	Serum	(131)
Sitatunga	UK	Feces	(125)
Sitatunga	South Korea	Feces	(122)
Water buck	UK	Feces	(125)
Waterbuck	USA (Ohio)	Feces	(82)
Water buffalo	Bulgaria	Serum	(132)
Water buffalo	Egypt	Feces	(133)
Water buffalo	Egypt	Feces	(134)
Water buffalo	Egypt	Feces	(135)
Water buffalo	Italy	Feces—intestinal contents	(136, 137)
Water buffalo	Bangladesh	Feces	(138)
Water deer	South Korea	Nasal swabs	(139)
White-tailed deer	USA (Ohio)	Feces	(19)
Wood bison	Canada	Serum	(140)
Wisent	South Korea	Feces	(122)
Yak	China	Feces	(141)

Complete genome sequencing revealed that some of the bovine-like CoVs isolated from wild and captive ruminants (giraffe, sambar deer, white-tailed deer, waterbuck, water deer, sable antelope, camelids and water buffalo) share high (>98–99.6%) sequence identity with enteric and respiratory BCoV strains, supporting their close genetic relatedness (19, 48, 84). Similar to the lack of genetic markers for discriminating between enteric and respiratory BCoVs, no reliable genetic markers were identified to distinguish between BCoVs and ruminant bovine-like CoVs (19). Instead, phylogenetic analysis demonstrates clustering of BCoVs/bovine-like CoVs according to the year of detection/isolation (Figure 2), which suggests that there is likely a co-evolution with continuous exchange by the respective virus pools. Finally, no consistent changes were observed in the genomes of bovine-like CoVs after passage in gnotobiotic calves. These data suggest that wild ruminants may represent a natural reservoir for bovine-like CoVs and transmit them to cattle or vice versa; these BCoVs represent a single host-range CoV species (27). Such interspecies infections may result in generation of more genetically divergent, potentially recombinant strains that escape immune responses and could potentially spread to other species including humans based on an historical precedent (42). Further, similar to BCoVs, a number of bovine-like CoVs from wild/captive ruminants can readily replicate in human rectal tumor-18 (HRT-18) cells (19, 48, 82), which further suggests a possibility for zoonotic transmission events.

Of interest, while bovine-like CoVs are frequently detected in diarrheic or healthy wild ruminants, there are no reports of occurrence of CoV-related respiratory disease outbreaks in the wild ruminants. This once again emphasizes the multifactorial nature of BRDC in cattle and suggests that its development is likely related to existing herd management practices including crowded housing, transportation, constant influx of new animals, production-associated stresses in cows, etc.

### BCoV-Like Infections of Non-ruminant Species

In addition to a broad range of domestic and wild ruminants, CoVs genetically/antigenically similar to BCoV have been detected in dogs with respiratory disease (142) and in humans (17). An enteropathogenic BCoV strain caused a subclinical infection and seroconversion in experimentally infected dogs (83). A recent report demonstrated that the HECoV-4408 strain infects upper respiratory and intestinal tracts causing diarrhea and intestinal lesions and conferring complete cross-protection against the virulent BCoV DB2 strain in gnotobiotic calves (47). Finally, while the most common ancestor has not been identified, it is assumed that HCoV-OC43 has emerged in the human population at the end of the 19th century likely originating form a BCoV ancestral strain via recombination events (42). Thus, detailed investigation of the endemic and emerging BCoV strains in the context of the host glycobiology is needed to identify and control potential pre-pandemic variants.

## Diagnosis

Pneumoenteric BCoVs replicate in the upper respiratory and the intestinal tracts and are detected in nasal secretions and feces, with nasal often preceding fecal shedding (27, 90, 95, 100, 101, 143). BCoV has also been detected or isolated from lung in animals with respiratory disease (88). The complete list of post-mortem diagnostic samples from animals with suspected respiratory (or enteric) BCoV disease includes tissues from the respiratory tract (e.g., nasal, pharyngeal, tracheal and lung tissues) (34, 89, 93) and from the distal small intestine and colon (101). For live animals, nasal secretions collected with sterile polyester swabs and feces collected in sterile fecal cups should be chilled and transported to the diagnostic lab (95, 144). From live calves with acute respiratory disease, tracheobronchial lavage fluids that were previously shown to be positive for BCoV antigen by ELISA can be aspirated (145).

The acute transient nature of enteric BCoV infections in younger calves and seronegative cattle necessitates sample collection at disease onset or shortly thereafter. However, persistent or long-term intermittent shedding has been reported in recovered or healthy cattle, respectively (59, 60, 146). Due to the stress of shipping and comingling of animals from different sources, the peak of BCoV nasal (or fecal) shedding associated with BRDC infections occurs within 1 week after arrival to the feedlot (89–91, 95).

BCoV infection can be diagnosed by detection of virus, viral antigen, or viral RNA in tissues, or various animal secretions/excretions. Comprehensive diagnosis includes virus detection in nasal secretions, lung homogenates or feces, and virus isolation in HRT-18 cells during the acute phase and/or detection of BCoV-binding or virus neutralizing (VN) antibodies in the convalescent phase (27, 89, 93, 144, 147).

Immunofluorescent/immunohistochemical staining with hyperimmune antiserum or MAbs to BCoV is performed for viral antigen detection in respiratory (trachea, lung) or intestinal (ileum, colon) tissues (frozen or paraffin-embedded). Detection of BCoV in nasal secretions or feces can be done using immune electron microscopy, which has the advantage of detecting other viruses, but its sensitivity is relatively low (59, 82). BCoV antigens are commonly detected by ELISA using BCoV MAbs which can be used for fast and reliable analysis of large sample numbers. RT-PCR, nested RT-PCR, real-time qRT-PCR (targeting most conserved genomic regions—-ORF1ab or N gene) are the most sensitive assays currently available for BCoV detection (45, 146, 148). For fecal samples, internal controls or additional sample dilution may be needed to detect interference by PCR inhibitors (149).

Antibodies to BCoV in serum, nasal secretions, or feces can be quantitated in ELISA [overall or isotype-specific antibodies (IgM, IgA, IgG1, and IgG2)] or using VN or HI tests that measure functional neutralizing or hemagglutinating antibodies, respectively (34, 150, 151). Because BCoV antibodies are widespread in cattle, paired acute and convalescent serum samples are needed for serologic diagnosis of BCoV infections (26).

## Immunity, Vaccines, and Other Prevention Strategies

The correlates of immune protection against BCoV infections remain poorly defined. In multiple studies the BCoV-binding, neutralizing and HI antibody levels in serum of naturally infected calves or cattle on arrival in feedlots were correlated with protection against enteric or respiratory disease (including pneumonia), or BCoV shedding (27, 91, 94, 95, 98, 151–153). Inoculation of gnotobiotic or colostrum-deprived calves with CD, WD, or respiratory BCoV strains induced complete protection against diarrhea following challenge with a CD strain (43, 45). However, whether the serum antibodies themselves confer protection against BCoV or they only reflect previous BCoV exposure is uncertain and requires further investigation. Limited data from some epidemiologic studies of BRDC infections in cattle suggest that BCoV serum antibody levels may be a marker for respiratory protection (26). So, Lin et al. demonstrated that cattle shedding respiratory BCoV at the start of the epizootic of BRDC/pneumonia had low levels of VN and HI antibodies, whereas cattle with high levels of antibodies against the HE and S viral glycoproteins remained negative for respiratory BCoV (151, 152). Furthermore, the levels of VN, HI and IgM, IgG1, and IgG2-BCoV antibody levels in serum were highly correlated with protection against respiratory BCoV infection. Finally, in cattle with fatal respiratory BCoV infections, only IgM antibody responses were detected. However, a recent longitudinal study demonstrated that BCoV serum antibody levels did not correlate with the incidence of BRDC or BCoV shedding (107).

Optimal vaccines against pneumoenteric mucosal pathogens should be delivered to and be effective at both sites of virus replication (respiratory and intestinal tracts) to provide optimal protection. Also, most vaccines against mucosal pathogens fail to induce sterilizing immunity or to prevent subsequent reinfections, as observed for natural or experimental BCoV infections (45, 59). Thus, the goal of vaccination is to confer broad protection against the severe enteric/respiratory disease that may lead to mortality and requires treatments. These objectives may be best accomplished by vaccinating calves on farms prior to shipping to auction barns or feedlots because BCoV infections occurring at feedlots necessitate rapid onset of protective immune responses. In support of this hypothesis, a recent study demonstrated that IN vaccination of feedlot calves with a modified live-BCoV calf vaccine on entry to a feedlot reduced the risk of treatment for BRDC in calves (99). These data confirm the results of experimental challenge studies of calves confirming cross-protection among BCoV strains of distinct clinical origin. Another recent study from Uruguay demonstrated that maternal vaccination against BCoV reduced BCoV shedding in their calves (154). Further studies are needed to determine whether inclusion of a mixture of CD/WD/BRDC strains could be an optimal strategy to develop a single broad-spectrum BCoV vaccine effective against BCoV infections associated with distinct clinical syndromes (26).

While several licensed modified live vaccines against BCoV are currently available (Bovilis, Merck/PBS Animal Health; Guardian, Merck; Calf-Guard, ScourGuard 4KC and BoviShield Gold 5, Zoetis; CattleWin BC, KyotoBiken Laboratories, Kyoto, Japan), observational data or field studies evaluating their protective efficacy against CD/WD remain scarce, while some of studies suggest they induce poor protection against BRDC viruses (155). Additionally, most studies evaluating candidate BCoV vaccines report on their safety and immunogenicity but not protective efficacy (156–159).

The ubiquitous nature of BCoV infections, lack of comprehensive data on vaccine efficacy, short duration of post-vaccine mucosal immunity, immunological immaturity of calves, and potential interference by maternal antibodies represent some of the existing challenges that likely contributed to the lack of optimal protective effects against BRDC and BCoV diarrheal disease. Moreover, the complexity of vaccine management (the need to target variable age groups of cattle, production status, newly arriving animals, etc.) and suboptimal cost-benefit ratio of vaccine use suggest that alternative control and prevention strategies need to be evaluated and employed. So far, Norway is the only country that has been implementing a joint biosecurity control program for BCoV and BRSV since 2016 (160). This program has the goal of reducing the occurrence of BCoV on a herd level by implementing strict biosecurity measures and financial reward system for compliant farmers/industries, while vaccination is not part of this program. The efficacy of such approach remains to be determined.

## Concluding Remarks

An overview of the existing data suggests that additional research is needed to understand the basic mechanisms of BCoV respiratory and enteric disease pathogenesis and the variables and interactions between viral, host and environmental factors that exacerbate disease or can lead to enhanced shedding and transmission. Further additional extensive research is needed to identify correlates of immune protection and attributes required to generate effective vaccines/regimens that can prevent severe disease and limit virus spread. The limited knowledge of the mechanisms that regulate BCoV interspecies transmission and determine broad host ranges warrants further research. Finally, continuous investigation is needed to better understand the ecology of bovine-like CoVs present in wildlife reservoirs (including wild ruminants or other susceptible species) or the threats they represent for public or animal health.

## Author Contributions

AV generated the initial draft. AV and LS have revised the final content critically. Both authors contributed to the article and approved the submitted version.

## Conflict of Interest

The authors declare that the research was conducted in the absence of any commercial or financial relationships that could be construed as a potential conflict of interest.
